# Effects of physical exercise on executive functions of individuals with schizophrenia spectrum disorders: Protocol for a systematic review and meta-analysis

**DOI:** 10.1371/journal.pone.0296273

**Published:** 2024-01-02

**Authors:** Nuria Pérez-Romero, Christian Campos-Jara, Caterina Pesce, Sergio Araya Sierralta, Enrique Cerda-Vega, Rodrigo Ramirez-Campillo, Rodrigo Campos-Jara, Cristian Martínez-Salazar, Cristián Arellano-Roco, Falonn Contreras-Osorio

**Affiliations:** 1 Exercise and Rehabilitation Sciences Institute, Postgraduate, Faculty of Rehabilitation Sciences, Universidad Andres Bello, Santiago de Chile, Chile; 2 Exercise and Rehabilitation Sciences Institute, Faculty of Rehabilitation Sciences, Universidad Andres Bello, Santiago de Chile, Santiago, Chile; 3 Department of Movement, Human and Health Sciences, University of Rome “Foro Italico”, Rome, Italy; 4 Departamento de Educación Física, Universidad de Atacama, Copiapó, Chile; 5 Exercise and Rehabilitation Sciences Institute, School of Physical Therapy, Faculty of Rehabilitation Sciences, Universidad Andres Bello, Santiago de Chile, Santiago, Chile; 6 Hospital Mauricio Heyermann, Servicio de Psiquiatría, Angol, Chile; 7 Department of Physical Education, Sports, and Recreation, Pedagogy in Physical Education, School of Education and Social Sciences and Humanities, Universidad de La Frontera, Temuco, Chile; 8 Laboratorio de Neuromecanica Aplicada, Escuela de kinesiología, Facultad de Ciencias Médicas, Universidad de Santiago de Chile, Santiago, Chile; Universidad de Málaga, SPAIN

## Abstract

**Introduction:**

Executive functions are commonly impaired in individuals with schizophrenia spectrum disorders. Physical exercise has the potential for improving executive functions and can be easily implemented as a therapeutic method. However, there are only few systematic reviews of exercise effects in schizophrenia including cognitive outcomes, and no meta-analytical syntheses of effects on “cool” and “hot” executive functions. The purpose of this systematic review and meta-analysis will be to determine the effects of physical exercise on “cool” and “hot” executive functions of adults with schizophrenia spectrum disorders.

**Methods and analysis:**

This protocol was guided by PRISMA-P guidelines. Studies will be searched using combinations of keywords and medical terms in the Web of Science, PubMed, Scopus, and EBSCO databases. Inclusion criteria will be determined as per PICOS approach. The risk of bias will be assessed using the Cochrane RoB2 tool. The certainty of evidence (per outcome) will be assessed using the GRADE method. The meta-analyses will be performed using the DerSimonian and Laird random effects model. Effect sizes (Hedges’ g) with 95% confidence intervals will be calculated for each main outcome.

**Conclusions:**

The results of this review may be useful for mental health professionals to design treatment plans for adults with schizophrenia spectrum disorders, offering potential benefits related to the quality of life and cognitive abilities of this population.

**PROSPERO registration number:**

CRD42023392295.

## Introduction

Schizophrenia is considered a devastating disease because of the associated social and neurocognitive disorders, which can prevent patients from achieving their professional and life goals [1–3]. This diagnosis is currently considered a spectrum since the release of the fifth version of the Diagnostic Statistical Manual (DSM-V) [4], which eliminates the conception of schizophrenia and its subtypes. This makes it possible to approach this condition in a more homogeneous and comprehensive way, as it considers the diversity both in the severity and persistence of its symptoms [5, 6] Schizophrenia spectrum is characterized by the presence of psychotic symptoms—e.g., vivid perceptions in the absence of an external stimuli (hallucinations), or correctly sensed stimuli that are given improper additional significance (delusional perceptions), and difficulty in keeping thoughts straight (disorganized thinking)—and, occasionally, abnormal awake states lacking movement and communication in response to other people and their environment (catatonia) [7].

In addition to general and other symptoms that are specific to the spectrum, schizophrenia is characterized by early cognitive impairments that are initially maximal for functions as verbal memory and lower-level cognition as information processing speed [8, 9] but expand, in chronic schizophrenia, into a broader array of higher-level cognitive functions including attention, problem solving, executive functions and social cognition [10–12]. In a recent umbrella review [13], 63 systematic reviews analyzing cognitive functions in individuals with schizophrenia were identified. The study [13] observed that those diagnosed with schizophrenia exhibit more pronounced cognitive impairment compared to both healthy controls and individuals with other affective disorders. This is especially notable in the areas of processing speed, verbal memory, and working memory, irrespective of their pharmacological treatment [13].

Executive functions are understood as a range of higher-order cognitive skills that allow for the supervision and control of emotions, behaviors, and other mental processes in voluntary tasks with processing demands that are directed to the analysis of information from multiple sources, decision making, goal achievement, or flexible response to environmental demands [14, 15]. Social cognition may be conceived as a ‘hot’ executive function that, compared to those of a more cognitive type (‘cool’ executive functions as cognitive flexibility), are of more socio-emotional type [16]. Indeed, social cognition entails domains of emotion processing and theory of mind, which are impaired in schizophrenia [17, 18]. Although ‘cool’ and ‘hot’ executive functions have their main substrate in different areas of the prefrontal cortex (dorsolateral and ventromedial, respectively), they share more neural networks than lower-level cognitive functions due to their higher-order nature. Thus, their impairment in schizophrenia is associated with more generalized structural brain abnormalities (e.g., brain regions with reduced volume) in more networks [10–12]. Cool and hot executive functions, along with attention skills and processing speed have been determined to be predictors of global functional outcome—including self-care, work outcomes, and social functioning—both in patients with early-onset psychosis and chronic schizophrenia [19, 20].

Neurocognitive impairments are reported to appear before other clinical symptoms become evident and can persist after they are control, showing little response to pharmacological treatment. They can also predict the onset of psychosis and mortality in schizophrenia spectrum disorders [10, 11, 21, 22]. Other factors that may further increase the mortality rates include the following: the presence of unhealthy lifestyles, which include unhealthy habits such as smoking, sedentary lifestyle, or alcohol abuse; associated medical conditions, e.g., cardiovascular diseases or metabolic abnormalities; and a high risk of suicide. These associated factors may reduce the life expectancy of the patients to 15–20 years lower than that of the general population [23–26].

People with schizophrenia are more sedentary than healthy controls of the same age and sex, a trend that is maintained when quantifying the mean daily physical activity of a moderate to vigorous intensity, where people with schizophrenia were found to be the least active (on average 37.5 min/day of moderate-to-vigorous physical activity, 95% confidence interval [CI]: 29.1–46.0) within this array of medical conditions [27]. These antecedents are of great importance when considering that young to late middle-aged adults with schizophrenia are 3.6-fold more likely to die from cardiovascular diseases as compared to the general population (mortality rate: 403.2 per 100,000 person-years) [28]. A sedentary lifestyle and a low level of physical activity among people with schizophrenia are also associated with worse cognitive performance [29]. Physical exercise interventions in this population can improve cognitive performance, and reduce negative and positive symptoms [27, 30, 31]. Cognitive improvements have been associated with an increase in hippocampal volume [32], an area usually affected from the early stages of schizophrenia [30, 33]. Additionally, physical exercise in people with schizophrenia may reduce use of medications, decreasing their side effects, and provide an additional therapeutic option with impact in areas of functioning where medications have been shown to provide only limited improvements (e.g., on impaired cognitive skills) [30, 34, 35].

Taken together, this evidence suggests that promoting interventions that aim to control modifiable cardiovascular risk factors (such as physical inactivity) become a public health priority for vulnerable populations like this one [36]. Indeed, physical activity and exercise can be used as primary or adjuvant therapy to improve clinical symptoms (positive, negative, and cognitive symptoms), quality of life, and global functioning, as well as neuroplasticity and neurogenesis [35, 37–42]. To address the therapeutic value of being active for individuals with schizophrenia spectrum disorder, the terms ‘physical activity’ and ‘physical exercise’ must be differentiated. The first refers to any bodily movement that involves energy expenditure through skeletal muscles [43]. Within this broader term, ‘exercise’ is a specific form of physical activity that is planned and structured, with the aim of improvement or maintenance not only of physical but also mental health. Furthermore, exercise interventions may consist of single bouts aimed at reaping transient benefits (acute exercise) or continuative practice over weeks to years aimed at reaping long-term effects (chronic exercise).

### Review rationale

In chronic exercise studies with people with schizophrenia, a relationship has been found between exercise and cognition as a function of the quantitative parameters of the exercise intervention (frequency, intensity, and duration) [37–39]. Among the different exercise modalities, major attention has been devoted, as traditionally in exercise-cognition research with healthy populations [44], to aerobic exercise also with persons affected by schizophrenia [30, 31, 37, 40, 45, 46]. Firth and colleagues [35] and Shimada and colleagues [33] reported that aerobic exercise causes significant improvements of medium to large size in global cognition, working memory, and attention. Firth et al. [35] also found large effects on social cognition, while Xu et al. [47] found no beneficial effects of aerobic exercise on working memory and attention, but only on other higher-level, ‘cool’ executive functions (reasoning and problem solving). However, those reviews neither provided a thorough analysis of the effects of physical exercise on executive functions, nor contrasted the effects on ‘cool’ and ‘hot’ executive functions that might further our understanding of the effect of physical exercise on more cognitive vs. more socio-emotional aspects of executive functioning.

Moreover, beyond the metabolic demands of aerobic exercise that may trigger neurobiological and -physiological mechanisms that explain the effects on brain health and cognitive functioning, also the qualitative characteristics of exercise, as the type of exercise, have attracted increasing attention in exercise-cognition research [48]. Both the general and specific benefits of physical exercise on cognition in patients diagnosed with schizophrenia have been studied in previous narrative and systematic reviews, covering and merging different types of exercise [30, 31, 37, 45, 46, 49], or looking at the efficacy of combined physical and cognitive training [49], or focusing specifically on aerobic exercise [30, 33, 35, 47], holistic movement practices (e.g., yoga) [50], and mindful exercise [51]. However, these reviews did not contrast the effects on executive functions of different types of exercise considering, beyond their quantitative metabolic demands (e.g., aerobic vs. resistance or high-intensity training), also their qualitative levels of cognitive challenge (e.g., soccer, mindful movements). Lastly, not only the quantitative and qualitative characteristics of the physical exercise tasks matter. There is emerging interest for the role of the exercise context, conceived not merely as the background of the exercise intervention, but also as a moderator of exercise effects on cognition [52] and, more broadly, of exercise effects on mental health [53]. For example, the outdoor environment may have stress-relief and attention restoration properties, and the group delivery or team play may trigger social interaction that amplify the efficacy of the exercise dose on executive function [52].

When considering the assumptions by Shojania et al. [54] regarding the speed with which systematic reviews become outdated (and, in this sense, 50% of them would become obsolete within 5 years in emerging areas such as mental health), is advisable to promote regular literature reviews so as to provide updated evidence that facilitates decision-making concerning the treatment of people with schizophrenia. In our case, only two previous systematic reviews addressed a similar topic in the last 5 years [33, 47]. However, these studies only focused on the effects of aerobic exercise on the cognitive domains established by the MATRICS Consensus Cognitive Battery (MCCB) [55, 56], without specifically analyzing two of the main dimensions of executive function (inhibitory control and cognitive flexibility).

Additionally, a novel approach in this systematic review will be the inclusion of the cognitive outcomes core executive functions (inhibition, working memory, cognitive flexibility) related to the "cool" executive functions, and also the inclusion of the outcome social cognition, emotion regulation, emotion recognition, and decision making, related to "hot" executive functions. Another novel approach in this systematic review will be the analyses of potential moderators (e.g., exercise frequency; exercise intensity; exercise duration; indoor/outdoor exercise; individual/group exercise; aerobic/anaerobic exercise) on the effect of physical exercise on executive functions.

In summary, this systematic review aims to provide a current perspective on how different types of exercises may impact the executive functions of individuals with schizophrenia, addressing limitations observed in previous studies. For instance, our protocol considers includable all forms of exercise, overcoming a significant constraint in prior reviews that limited their review to a specific/single form of exercise (e.g., aerobic; yoga). Additionally, our protocol considers separate analysis for "hot" and "cold" executive functions, deepening our understanding of their key underlying variables. This innovative approach will enhance the comprehension of exercise and its potential effects on cognitive and socioemotional aspects of executive functions, paving the way for more personalized (and potentially effective) therapies. Further, by exploring a diverse range of moderators (e.g., type of exercise, intervention duration, social support), the study seeks to offer specific recommendations for implementing exercise programs in clinical settings with a more holistic approach, marking a significant advancement in this field.

### Objectives

Therefore, the objective of the present study is to synthesize the scientific literature currently available on the effects of physical exercise concerning the ‘cool’ and ‘hot’ executive functions of adults diagnosed with schizophrenia spectrum compared with an active or passive control condition, through a systematic review with meta-analysis. Additionally, this study aims to analyze the effects of different characteristics of physical exercise (e.g., type, intensity, duration) and social moderators (e.g., level of social support, family structure, academic level) on different executive functions in population diagnosed with schizophrenia spectrum. It is anticipated that results from this systematic review would provide updated and relevant information for the potential generation of intervention programs aimed to positively influence higher order cognitive functions (cool and hot executive functions) closely linked to the functional performance and prognosis of people diagnosed with schizophrenia spectrum, facilitating therapeutic decision making [57, 58].

## Methods and analysis

This protocol was guided by PRISMA-P guidelines [59], and the methodology described in some sections of this protocol follows that performed by Contreras-Osorio et al. [60].

### Eligibility criteria

The inclusion and exclusion criteria are described using the PICOS strategy and are displayed in Table 1.

**Table 1:** Eligibility criteria.

	Inclusion	Exclusion
Population	Adult individuals (age, 18–60 years) diagnosed with schizophrenia spectrum according to the Diagnostic and Statistical Manual of Mental Disorders DSM-5 or its equivalents in DSM-I, II, III, III-R, IV, or IV-TR, or in the International Classification of Diseases (ICD-9, 10, 4).	Children, adolescents, or older adults. Patients with other major disorders, such as bipolar disorder or dementia. Patients with limited physical activity (e.g., cardiopulmonary disease). People with intellectual disabilities.
Intervention	Interventions involving physical exercise programs with a duration of at least four weeks, without restrictions regarding the type of exercise (e.g., multicomponent, aerobic, sports programs, or yoga).	Acute interventions (one single session) or interventions not related to physical exercise.
Comparator	Control group with the same diagnosis that is not exposed to the intervention program. It can be active (e.g., relaxation techniques) or passive (continuing with their daily activities or waiting list).	Absence of a control group.
Outcomes	Pre- and post-intervention data from at least one instrument of direct application of ‘cool’ or ‘hot’ executive functions that has been previously validated, (e.g., Trail Making Test, Flanker task, N-back task or Iowa gambling task).	Indirect evaluation measures of the executive function. Self-report questionnaires.
Study design	Randomized and non-randomized longitudinal studies.	Cross-sectional studies; case studies.

Physical exercise is defined as a planned, structured, and repetitive body movement aimed at improving or maintaining one or multiple outcomes (e.g., physical, cognitive) [61, 62], thus extending its effects to the field of mental health [63, 64]. The minimum duration of the intervention will be established according to previous literature, as cognitive improvements have been described after the fourth week for physical exercise interventions in people with schizophrenia [65]

The outcomes must be reported using previously validated instruments to assess ‘cool’ or ‘hot’ executive functions (e.g., working memory, inhibition, cognitive flexibility, decision-making, social cognition, emotion regulation or emotion recognition) in adults. Some examples of valid instruments: N-back task [66] to assess working memory, the Flanker task to assess inhibition [67], the Trail Making Test-Part B to assess cognitive flexibility [68], the Iowa gambling task to assess the affective decision-making ability [69] or the Non-verbal emotion recognition task to assess the accuracy of judging nonverbal emotions [70]. Studies that use indirect evaluation instruments—meaning that they evaluate the opinion of a third party regarding the user’s performance or self-report questionnaires—will not be considered.

### Sources of information

The review will include the following databases: PubMed, Web of Science, EBSCO, and Scopus. Studies that meet the eligibility criteria found through other sources (e.g., references from review studies found in databases) will also be incorporated. Finally, studies suggested by external experts (n = 2), chosen through the Expertscape ranking, will also be added. The experts will be selected using the terms "Executive function" in the link: https://www.expertscape.com/ex/executive+function. The search will begin after acceptance of the protocol for publication. Pilot tests of the search strategies were carried out in January of 2023.

### Search strategy

Two authors (F.C.-O. and N.P-R.) will carry out the systematic search, without restrictions to the sex of the participants, or the date of publication. The search for articles in the different databases will begin once the protocol has been accepted for publication. To do this, the above-mentioned search strategies will be applied using medical subject headings (MeSH) and free-text terms (Table A1 in S1 Appendix).

#### Selection process

The selection of the studies that comprise the review will be recorded using a flowchart, as established in the PRISMA guidelines [71], detailing the selection process and the corresponding reasons for exclusion. After the identification of the documents through databases, duplicates will be removed using a bibliographic reference management software package. Manual deletions are considered, if needed (N.P-R.). The titles and abstracts will be subsequently read, where two authors will appraise their eligibility (F.C.-O. and N.P.-R.). Reference lists of included articles and reviews identified during the search process will also be assessed to select potentially eligible studies. Should there be any discrepancies regarding the decision of the authors, they will be resolved by consensus with a third author (C.C.-J.).

### Data extraction and management

For each of the studies included, the following data will be identified: year of publication, author, sample size, characteristics of participants (sex, age, fitness level, psychiatric diagnosis and severity, comorbidities, pharmacological treatment, and social-related information [e.g., level of social support, family structure, academic level]), description of the physical exercise program, weekly frequency of the intervention, duration of the intervention (in weeks), duration (in minutes) and intensity of sessions and/or exercises (e.g., heart rate; Borg scale), dimensions of the executive function assessed (working memory, inhibition, and cognitive flexibility), tasks or test used for assessment (e.g., Trail Making Test-Part B [72] to assess cognitive flexibility, N-Back [66] to assess working memory, Stroop [67] to assess inhibition), and description of the control condition.

The means and standard deviation values of each outcome will be registered, considering the period before and after the intervention. Data extraction will be performed by one author (F.C.-O.) and will be verified by a second author (N. P.-R.). Each author will store the data in a Microsoft Excel spreadsheet (Microsoft Corporation, Redmond, WA, USA) independently. Any conflict will be resolved by consensus with a third author (R.R.-C.). Should the data not be clearly or completely contained in the document, the contacting procedures for the authors described in the previous literature will be applied [60, 73, 74]. In this sense, the authors of these studies will be contacted up to a maximum of two times within a period of two weeks. Should there be no answer, or a lack of data required, the study will be excluded from the analysis. When the data are represented in graphs or figures, two of the authors (FC-O. and NP-R.) will use a validated software (r = 0.99, p < 0.001) [75] (WebPlotDigitizer, version 4.5, Pacifica, CA, USA; https://apps.automeris.io/wpd/) to extract the numerical data necessary to calculate Cronbach’s Alpha. Should any discrepancies arise, these will be resolved by consensus with a third author (C.C.-J.).

### Risk of bias of individual studies

As in previous studies in the field [76–79], the risk of bias will be assessed using the second version of the Cochrane RoB tool (RoB2) [80] for randomized trials, classifying them as low, some concerns or high risk according to the analysis of five domains: randomization process, deviations from intended interventions, missing outcome data, measurement of the outcome, and selection of the reported result. The risk of bias will be evaluated by two authors (F.C.-O. and C.C.-J.) independently, resolving discrepancies with a third author (N.P.-R.). Regardless of the results derived after the application of the RoB2, all studies that meet the inclusion criteria will be considered for the analyses, although the interpretation and discussion of the outcomes will consider the RoB2 results.

In addition, the GRADE method (Grading of Recommendations, Assessment, Development and Evaluation) will be applied to synthesize and assess the certainty on the evidence for each outcome [81], with the categorization high, moderate, low, or very low [82, 83].

### Qualitative data synthesis

The key characteristics and important questions from the included studies in relation to the aim of this review will be summarized in tables. The characteristics of the participants for the included studies will be recorded, including the number of participants in experimental and control groups, sex (female/male), age (years), diagnosis (type; test; severity), baseline cognitive status, co-morbidities, and co-treatments (e.g., medication). The main characteristics of the interventions (and control condition) implemented in the included studies will also be recorded: description of the exercise program (e.g., type of exercises), level of compliance with the intervention program (e.g., percentage of total sessions completed; average number of sessions completed), total duration of intervention (weeks), weekly training frequency, duration of each session (minutes), intensity (e.g., percentage of maximum heart rate; percentage of heart rate reserve), test used to measure executive function (e.g., flanker task; Iowa gambling task).

### Meta-analysis

Whether if a meta-analysis on a given outcome is possible or not will be determined when data have been extracted. It is expected to analyze the outcomes i) “cool” executive functions, also involving the isolated analysis for working memory, inhibition, and cognitive flexibility, and ii) “hot” executive functions, also involving the isolated analysis for social cognition, decision making, emotion regulation, and emotion recognition. At least three studies will be required to perform the meta-analysis per each outcome measure [84, 85] due to the usually low number of participants in the studies published in this area [86]. The Hedges’g effect size (ES) will be estimated with 95% confidence interval (CI) and its prediction interval. The ES will be estimated based on the mean and standard deviation before and after the intervention period in the experimental groups compared to the control. Should these studies present data other than mean values and/or standard deviation, appropriate statistical conversions will be performed prior to meta-analysis. The standard deviation after the intervention will be used to standardize the data. The DerSimonian and Laird methods will be applied for the meta-analysis.

The estimated ES will be assessed using the following categorization: <0.2 trivial, 0.2–0.6 small, >0.6–1.2 moderate, >1.2–2.0 large, >2.0–4.0 very large, and >4.0 extremely large [87]. Of note, outlier ES values were sought to be identified. An outlier is an extreme case that seems to be well separated from the rest of the data [88] and in a meta-analysis or meta-regression can affect its validity and robustness [89], and the interpretations, conclusion and inferences derived from it. Although there is no a single method to identify an outlier study [88], in physical exercise intervention studies, ES values ≥3.0 (i.e., improvement of ≥3 standard deviations from the mean) are unlikely after most interventions, and thus will be considered outliers [88].

In studies including more than one intervention group and one single control group, the sample size of the control group will be divided proportionally to facilitate comparison between several groups. The degree of heterogeneity will be assessed using the I^2^ statistic, with values of <25%, 25–75% and >75% which indicate low, moderate, and high levels, respectively [83].

The risk of publication bias will be assessed for continuous variables (≥10 studies per outcome) using Egger’s test [79, 90]. To adjust for publication bias, a sensitivity analysis will be performed using the trim-and-fill method [91], with L0 being the default estimator for the number of missing studies [92]. A multivariate DerSimonian and Laird random-effects model meta-regression will be conducted to verify if any of the continuous moderators (e.g., training frequency; training duration; total number of intervention sessions) explained the effects of interventions on the dependent variables. The computation of meta-regression will be performed with at least 10 studies per covariate [93]. Additionally, a sensitivity analyses will be performed to assess the robustness of the summary estimates (e.g., p-value, ES, I^2^). To examine the effects of each result from each study on the overall findings, results will be analysed with each study deleted from the model (automated leave-one-out analysis). All analyses will be performed using the Comprehensive Meta-Analysis software (version 2, Biostat, Englewood, NJ, USA). Statistical significance will be established at p ≤ 0.05.

### Moderators

Potential moderators [33, 94–100] on the response of executive functions to exercise will be analyzed, including: exercise intensity, intervention duration (weeks), weekly training volume (minutes), type of exercise (e.g., aerobic exercise; resistance exercise; High Intensity Interval Training; yoga; multicomponent), combined (successive) or integrated (dual task) physical and cognitive training, outdoor/indoor exercise, and individual/group/team exercise mode (i.e., lowest to highest social interaction level). Social moderators on the response of executive functions to exercise will also be considered as level of social support, family structure, or academic level [11, 101].

Where appropriate, analyses will be split using the median split technique. The median will be calculated when at least three studies provide data for a given moderator (for each of their categories). When two experimental groups with the same information for a given moderator are included in a study, only one of the groups will be considered to avoid undue influence in the calculation of the median. Furthermore, to minimize heterogeneity, median values will be calculated using only those studies that provide data for the outcome analyzed instead of using an overall median value for a given moderator.

## Discussion

The aim of this systematic review is to analyze the effects of physical exercise intervention programs on ‘cool’ and ‘hot’ executive functions (e.g., working memory, inhibition, cognitive flexibility, social cognition, decision making, emotion regulation and/or emotion recognition skills) of adults diagnosed with schizophrenia spectrum, in comparison with an active or passive control condition. The information derived from this systematic review (and metanalysis) could be useful for mental health professionals who plan to establish rehabilitation treatments for patients with a diagnosis related to schizophrenia and its spectrum, thus contributing to improve their quality of life and prognosis [33, 37].

According to the World Health Organization [102], schizophrenia is related to a high level of stigma and difficulty in receiving psychological care. Therefore, analyzing the benefits offered by other therapeutic options, such as physical exercise programs, contributes to increasing access to low-cost alternative treatments. Moreover, physical exercise can even reduce the side effects of medication, while improving health and reducing mortality in patients with this type of disorder [37]. However, regarding its effect on cognitive abilities, the scientific community concludes that further research is necessary, as mixed outcomes have been described [33, 35, 50, 51].

It should be mentioned that there is an absence of systematic reviews that specifically analyze the effects of physical exercise programs on the "cool" and “hot” executive functions. This is a gap that the present study intends to cover, and which is related to the importance of these cognitive skills in the daily functioning of adults with schizophrenia [103]. However, the limitations that arise because of the analysis of the outcomes will be studied to offer possible solutions and future related lines of research that contribute to enriching scientific knowledge.

The adaptive response of executive functions to exercise will be analysed as per possible moderators, including quantitative and qualitative characteristics of the exercise, but also in relation to the characteristics of the context (e.g., exercise performed as individual/group/team) and social moderators (e.g., level of social support, family structure, academic level). This would allow a new perspective of analysis, that incorporates the socio-emotional characteristics of the training programs. Additionally, the methodological evaluation of the selected studies, will offer relevant information that should be considered to improve the quality of future research. Finally, a peer-reviewed scientific journal will be considered for the publication of this systematic review and the final outcomes will be disseminated with the greatest possible social outreach, mainly focusing on professionals and patients related to schizophrenia spectrum.

In summary, this research stands out for its updated synthesis and planned quantitative assessment of the impact of physical exercise on executive functions in adults diagnosed with schizophrenia spectrum disorders. It goes beyond previous systematic reviews by including variables (e.g., ’hot’ executive functions) and moderators (e.g., characteristics of the context, social moderators) that were overlooked in the existing literature. Additionally, this systematic review will analyze recently published studies, which were not considered in prior systematic reviews. Indeed, the rapid rate of published scientific articles in the field suggest the need for an update. For example, a rapid search in PubMed with the terms "schizophrenia"[All Fields]) AND ("exercise"[All Fields]) retrieved 1,141 documents, and >50% of these documents were published between 2016–2023. Additionally, the study evaluates the impact of physical exercise across all its forms, addressing a limitation pointed out by Shimada et al. [33]. Moreover, it delves into both ’cool’ and ’hot’ executive functions, providing a thorough analysis of cognitive and socioemotional aspects with a more comprehensive perspective on the subject. This innovative approach, not extensively explored in prior literature, has the potential to offer a more complete understanding of the mechanisms behind the effects of exercise in this population, thereby aiding in therapeutic planning. Furthermore, considering moderators related to intervention characteristics, context, and social aspects may shed light on result heterogeneity and provide more targeted recommendations for implementing exercise programs in real clinical settings.

## Supporting information

S1 Checklist PRISMA-P 2015. (DOCX)

S1 Appendix Terms used in the search strategy according to each database. (DOCX)
