# Supplementary material for: Effects of physical exercise on executive functions of individuals with schizophrenia spectrum disorders: Protocol for a systematic review and meta-analysis
Source: PLoS One. 2024 Jan 2;19(1):e0296273. doi: 10.1371/journal.pone.0296273 (PMC10760781; doi:10.1371/journal.pone.0296273)
Supplement: S1 Appendix — (DOCX) [file pone.0296273.s002.docx]

**Annex A**

**Table A1. Terms used in the search strategy according to each database.**

| Database | Search strategy |
| --- | --- |
| EBSCO | AB (“executive function*” OR “cognitive function*” OR cognition OR “inhibitory control” OR “inhibition” OR “interference control” OR “executive control” OR “working memory” OR “updating” OR “cognitive flexibility” OR “shifting” OR “switching” OR “social cognition” OR “emotion regulation” OR “emotion recognition” OR “decision making”) AND AB (sport OR “modified sport” OR fitness OR exercise OR “physical activity” OR athletics OR "resistance training" OR “sport practice” OR “mindful movements” OR “mindfulness practice*” OR “yoga” OR “team game” OR “soccer”) AND AB (schizoph* OR catatonia OR "schizoaffective disorder" OR "schizophreniform disorder" OR "schizoid personality disorder" OR "psychotic disorder" OR "schizophrenia spectrum and other psychotic disorders") |
| Pubmed | (((((((((("executive function*"[Title/Abstract]) OR ("cognitive function*"[Title/Abstract])) OR (cognition[Title/Abstract])) OR ("inhibitory control"[Title/Abstract])) OR inhibition[Title/Abstract])) OR “interference control”[Title/Abstract])) OR “executive control”[Title/Abstract])) OR ("working memory"[Title/Abstract])) OR ("updating"[Title/Abstract])) OR ("cognitive flexibility"[Title/Abstract])) OR (shifting[Title/Abstract])) OR (“switching”[Title/Abstract])) OR (“social cognition”[Title/Abstract])) OR (“emotion regulation”[Title/Abstract])) OR (“emotion recognition”[Title/Abstract])) OR (“decision making”[Title/Abstract])) AND ((((((((sport[Title/Abstract]) OR ("modified sport"[Title/Abstract])) OR (fitness[Title/Abstract])) OR (exercise[Title/Abstract])) OR ("physical activity"[Title/Abstract])) OR (athletics[Title/Abstract])) OR ("resistance training"[Title/Abstract])) OR ("sport practice"[Title/Abstract]) OR ("mindful movements"[Title/Abstract])) OR ("mindfulness practice"[Title/Abstract])) OR ("yoga"[Title/Abstract])) OR ("team game"[Title/Abstract])) OR ("soccer"[Title/Abstract]))) AND (((((((schizoph*[Title/Abstract]) OR (catatonia[Title/Abstract])) OR ("schizoaffective disorder"[Title/Abstract])) OR ("schizophreniform disorder"[Title/Abstract])) OR ("schizoid personality disorder"[Title/Abstract])) OR ("psychotic disorder"[Title/Abstract])) OR ("schizophrenia spectrum and other psychotic disorders"[Title/Abstract])) |
| Scopus | (("executive function*") OR ("cognitive function*") OR (cognition) OR ("inhibitory control") OR (inhibition) OR (“interference control”) OR (“executive control”) ("working memory") OR ("updating") OR ("cognitive flexibility") OR (shifting) OR (switching) OR (“social cognition”) OR (“emotion regulation”) OR ("emotion recognition”) OR ("decision making”)) AND ((sport) OR ("modified sport") OR (fitness) OR (exercise) OR (“physical activity") OR (athletics) OR (“resistance training”) OR (“sport practice”) OR (“mindful movements”) OR ("mindfulness practice”) OR (yoga) OR (“team game”) OR (soccer)) AND ((schizoph*) OR (catatonia) OR ("schizoaffective disorder") OR ("schizophreniform disorder") OR ("schizoid personality disorder") OR ("psychotic disorder") OR ("schizophrenia spectrum and other psychotic disorders")) |
| Web of Science | ((TS = (“executive function*” OR “cognitive function*” OR cognition OR “inhibitory control” OR inhibition OR “interference control” OR “executive control” OR “working memory” OR “updating” OR “cognitive flexibility” OR shifting OR switching OR “social cognition” OR “emotion regulation” OR “emotion recognition” OR “decision making”)) AND TS = (sport OR “modified sport” OR fitness OR exercise OR “physical activity” OR athletics OR ""resistance training" OR “sport practice” OR “mindful movements” OR “mindfulness practice” OR yoga OR “team game” OR soccer)) AND TS = (schizoph* OR catatonia OR "schizoaffective disorder" OR "schizophreniform disorder" OR "schizoid personality disorder" OR "psychotic disorder" OR "schizophrenia spectrum and other psychotic disorders") |
